# Supplementary material for: Deubiquitinating Enzyme USP7 Is Required for Self-Renewal and Multipotency of Human Bone Marrow-Derived Mesenchymal Stromal Cells
Source: Int J Mol Sci. 2022 Aug 4;23(15):8674. doi: 10.3390/ijms23158674 (PMC9369338; doi:10.3390/ijms23158674)
Supplement: Supplementary file 1 [file ijms-23-08674-s001.zip › ijms-1828173-supplementary.pdf]

Supplementary Materials

Deubiquitinating enzyme USP7 is required for self-renewal and multipotency of human bone marrow-derived mesenchymal stromal cells

You Ji Kim [1,2,†], Kwang Hwan Park [1,†], Kyoung-Mi Lee [1], Yong-Min Chun [1,*], and Jin Woo Lee [1,2,*]

[1] Department of Orthopedic Surgery, Yonsei University College of Medicine, 50-1 Yonsei-ro, Seodaemun-gu, Seoul 03722, South Korea; no11252004@naver.com (Y.J.K.); KHPARK@yuhs.ac (K.H.P.); OXY002@yuhs.ac (K.M.L.)

[2] Brain Korea 21 PLUS Project for Medical Sciences, Yonsei University College of Medicine, 50-1 Yonsei-ro, Seodaemun-gu, Seoul 03722, South Korea

* **Correspondence**: ljwos@yuhs.ac (J.W.L.); Tel.: +82-2-2228-2190/ Fax: +82-2-363-1139, osmin120@yuhs.ac (Y-M.C.); Tel.: +82-2-2228-2190/ Fax: +82-2-363-1139.

† These authors contributed equally to this study.

E-mail address: ljwos@yuhs.ac (Jin Woo Lee), osmin120@yuhs.ac (Yong-Min Chun).

Supplemental methods

2.1. Chemical treatment of hBMSCs

The USP7 inhibitor, GNE6776 (5439-DK-010, R&D Systems, Minneapolis, MN, USA) was used at a concentration of 10 μg/mL.

2.2. Cell viability assay

hBMSCs were seeded in 12-well plates at a density of 1×10^4 cells/well. Cells were treated with the USP7 inhibitor at different doses for 48 h. 20 μl of EZ-Cytox solution was then added and cells were incubated at 37°C for 3 h. The spent medium was then transferred to 96-well plates and absorbance was recorded at 450 nm. All experiments were performed in triplicate.

Supplemental Figure S1. Effects of USP7 knockdown on the proliferation and colony-forming capacities of P4 cells.

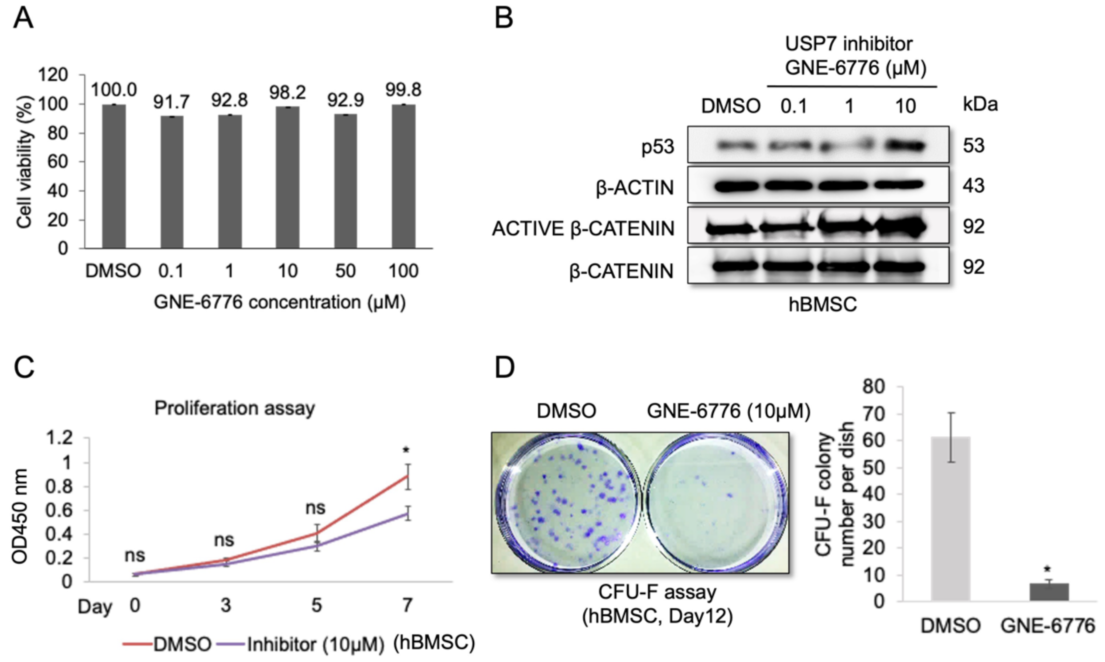

(A) qRT-PCR results after transfection with a negative control or USP7 siRNA. (B) Immunoblot results. (C) EZ-Cytox cell viability analysis results. (D) Colony-forming unit fibroblast assay (CFU-F) results. On day 12 of the CFU-F assay, colonies were stained with crystal violet. Numeric results are presented as mean ± SD. ns, not significant. * $P < 0.05$, ** $P < 0.01$, *** $P < 0.001$. $n = 3$.

Supplementary Table S1. Antibodies used for western blotting and immunoprecipitation

Antibody	
USP7	1:1000, D17C6, Cell Signaling Technology
β-actin	1:1000, sc-47778, Santa Cruz Biotechnology
HSP90	1:1000, sc-13110, Santa Cruz Biotechnology
Ki67	1:1000, sc-15402, Santa Cruz Biotechnology
p53	1:1000, sc126, Santa Cruz Biotechnology
p21	1:1000, sc6246, Santa Cruz Biotechnology
p16	1:1000, ab108349, Abcam
SOX2	1:1000, ab171380, or ab97959, Abcam
NANOG	1:1000, D73G4, Cell Signaling Technology
RUNX2	1:1000, sc390351, Santa Cruz Biotechnology
OPN	1:1000, sc-20788, Santa Cruz Biotechnology
FABP4	1:1000, sc-18661, Santa Cruz Biotechnology
COL2A1	1:1000, sc-518017, Santa Cruz Biotechnology
SOX9	1:1000, sc-166505, Santa Cruz Biotechnology
FLAG	1:1000, F1804, Sigma
GFP	1:1000, nb600-308, Novus Biologicals
Ubiquitin	1:1000, sc-8017, Santa Cruz Biotechnology
IgG rabbit control	cat. no. 12-370, Millipore
IgG mouse control	cat. no. 12-371, Millipore
β-catenin	1:1000, sc-7199, Santa Cruz Biotechnology
active β-catenin	1:1000, D13A1, Cell Signaling Technology

Supplementary Table S2. Primary sequences used for qRT-PCR.

Genes	Category	Sequence (5′ → 3′)
USP7	Forward	GTCACGATGACGACCTGTCTGT
	Reverse	GTAATCGCTCCACCAACTGCTG
18srRNA	Forward	ACACGGACAGGATTGACAGA
	Reverse	GCCAGAGTCTCGTTCGTTAT
GAPDH	Forward	P267613 (Bioneer)
	Reverse	
P53	Forward	CCTCAGCATCTTATCCGAGTGG
	Reverse	TGGATGGTGGTACAGTCAGAGC
SOX2	Forward	GCTACAGCATGATGCAGGACCA
	Reverse	TCTGCGAGCTGGTCATGGAGTT
NANOG	Forward	CTCCAACATCCTGAACCTCAGC
	Reverse	CGTCACACCATTGCTATTCTTCG

Supplementary Table S3. siRNA sequences

Scrambled negative control (siRNA cat. no. SN1003) and

USP7 siRNA (siRNA no. 7874-1)

Genes	Category	Sequence (5′ → 3′)
Negative control	Forward	CCUACGCCACCAAUUUCGU
	Reverse	ACGAAAUUGGUGGCGUAGG
USP7	Forward	CGACACUGCACUAAUGCU
	Reverse	AAGCAUUAGUGCAGUGUC
